# An Enhancement of Outdoor Location-Based Augmented Reality Anchor Precision through VSLAM and Google Street View

**DOI:** 10.3390/s24041161

**Published:** 2024-02-09

**Authors:** Komang Candra Brata, Nobuo Funabiki, Yohanes Yohanie Fridelin Panduman, Evianita Dewi Fajrianti

**Affiliations:** 1 Graduate School of Natural Science and Technology, Okayama University, Okayama 700-8530, Japan; p8f01q6f@s.okayama-u.ac.jp (Y.Y.F.P.); p2mu1tom@s.okayama-u.ac.jp (E.D.F.); 2 Department of Informatics Engineering, Universitas Brawijaya, Malang 65145, Indonesia

**Keywords:** location-based, augmented reality, SLAM, cloud-based matching, Android

## Abstract

Outdoor *Location-Based Augmented Reality (LAR)* applications require precise positioning for seamless integrations of virtual content into immersive experiences. However, common solutions in outdoor LAR applications rely on traditional smartphone sensor fusion methods, such as the *Global Positioning System (GPS)* and compasses, which often lack the accuracy needed for precise AR content alignments. In this paper, we introduce an innovative approach to enhance *LAR* anchor precision in outdoor environments. We leveraged *Visual Simultaneous Localization and Mapping (VSLAM)* technology, in combination with innovative cloud-based methodologies, and harnessed the extensive visual reference database of *Google Street View (GSV)*, to address the accuracy limitation problems. For the evaluation, 10 *Point of Interest (POI)* locations were used as anchor point coordinates in the experiments. We compared the accuracies between our approach and the common sensor fusion LAR solution comprehensively involving accuracy benchmarking and running load performance testing. The results demonstrate substantial enhancements in overall positioning accuracies compared to conventional GPS-based approaches for aligning AR anchor content in the real world.

## 1. Introduction

*Location-Based Augmented Reality (LAR)* applications have gained significant prominence in recent years due to the potential to deliver immersive and contextually relevant experiences [1]. The main goal of *LAR* is to link physical location coordinates with data processing environments, which allows a user to see the *Point of Interest (POI)* annotation of a particular landmark in a real environment. The use of *LAR* is particularly beneficial in education, entertainment, and navigation scenarios, such as pedestrian navigation in a particular venue [2,3,4]. The *LAR* application mainly relies on the positioning of the virtual content in the physical world to create a seamless blend of digital and real environments. The key metrics of *LAR* accuracy consist of the heading, vertical position, and horizontal position of the user’s device [5,6].

To provide augmented reality features, common *LAR* solutions often use traditional sensor fusion approaches, such as the *Global Positioning System (GPS)* and *Inertial Measurement Unit (IMU)*, which are embedded in smartphones to determine device locations and orientations to achieve immersive *LAR* experiences [7,8,9]. However, GPS sensors and compasses have limited accuracy. Hence, their accuracies often fall short of providing seamless and immersive *LAR* experiences, especially in dynamic outdoor environments [10]. Inaccurate AR anchor point information could be confusing to users, resulting in poor user experiences.

**Figure 1:**
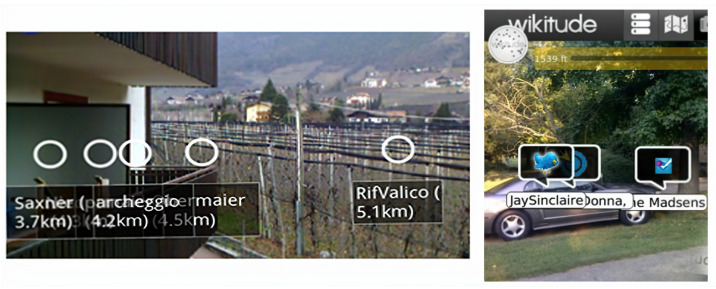
Common misalignments of anchor points in outdoor location-based AR.

The study by Uradzinski et al. highlighted the challenges of GPS-based systems in providing accurate location data, particularly in dynamic outdoor settings [11]. Similarly, other researchers emphasized the need for more advanced solutions to ensure the precise positioning of AR content, especially in outdoor environments with dynamic user movements [12,13]. Figure 1 illustrates an example of common drifted and inaccurate outdoor *LAR* anchor point positions.

In recent years, *Simultaneous Localization and Mapping (SLAM)* has seamlessly integrated into the augmented reality implementation, offering a solution to address the outdoor positioning challenges encountered by *LAR* [14,15,16]. In this regard, *Visual Simultaneous Localization and Mapping (VSLAM)* enhances the capabilities of traditional SLAM by incorporating visual data from camera feeds, making it especially well-suited for augmented reality applications in smartphones compared to other *SLAM* techniques [17,18,19].

However, most of the existing *VSLAM*-based solutions are implemented for indoor scenarios. Although the implementation of *VSLAM* in an outdoor *LAR* scenario is still limited, previous work by Liu et al. [20] and Tang et al. [21] have shed light on the potential of *VSLAM* in outdoor *LAR* applications, highlighting its capability to offer precise and robust localization and mapping. In addition to the possibility of improving localization performance, the *VSLAM* technique alone has several limitations, such as particular tracking loss and accumulating errors for building and localizing trajectory maps. Tracking losses are typically caused by large and fast changes in the camera position while walking. The lack of overlaps between consecutive reference image frames makes it difficult to extract sufficient features needed for building the trajectory map. Localization errors due to the erroneous calculation of location accumulate in the absence of feature matching [22].

Previously, we developed an outdoor *LAR* application that integrates *VSLAM* on top of the sensor fusion approach. The *ARCore* engine version 1.31 was used in this implementation. *ARCore*, also known as *Google Play Services for AR*, is a software development kit developed by *Google* that provides the AR generation process library and is the most compatible AR environment for *Android* development [23]. Powered by the *ARCore* library, this prototype aligns the generated AR objects with the user’s pose and surface. Unfortunately, the current implementation of this *LAR* application needs a large number of image references for optimal user localization tracking.

In this paper, we present an approach to enhance the precision of *LAR* anchors in outdoor environments by leveraging the *VSLAM* technique and *Google Street View (GSV)* on top of smartphone sensor fusion. As a well-known imagery location service, *GSV* provides a fast amount of imagery references of various outdoor locations worldwide [24]. Coupled with innovative cloud-based methodologies and harnessing the vast visual reference database offered by *GSV*, this research endeavors to address the accuracy constraints encountered by conventional methods. Additionally, the study by Campbell et al. in [25] emphasized the significance of cloud-based techniques and *GSV* for improving spatial recognition. These research findings collectively underscore the importance of integrating *VSLAM*, cloud-based matching, and visual references, as explored in this paper.

Through comprehensive quantitative evaluations, including benchmark testing and load performance testing, this paper investigates the feasibility of the proposed approach. The results indicate that the proposed approach significantly enhances the accuracy of AR anchor positions, whereas the combination of *VSLAM* and cloud-based matching from *GSV* imagery in the proposal requires additional computation resources.

The remainder of this article is organized as follows: Section 2 introduces the related work. Section 3 describes our previous work in this domain. Section 4 presents the key technologies, the prototype design and implementation, and the evaluation method of the proposal. Section 5 shows the results and discusses their analysis. Finally, Section 6 provides conclusions with future work.

## 2. Related Work in the Literature

In this section, we provide an overview of key findings from related work that has paved the way for the proposed approach presented in this paper.

### 2.1. Sensor Fusion LAR Methods

By building upon the knowledge and insights gained from prior research, our approach aims to advance the state of outdoor *LAR* technology and open new possibilities for the development of seamless and engaging outdoor location-based AR experiences.

In [26], Huuskonen et al. explored *LAR* solutions in precision farming and highlighted the potential of *LAR* to revolutionize agriculture. By utilizing the *GPS* and sensor fusion approaches for augmented reality features, their system automatically determines the locations for soil samples based on a soil map created from drone images after plowing and a wearable augmented reality technology to guide the user to the generated sample points. This work contributes to the transformative impacts of agricultural technologies on resource-efficient practices in southern Finland.

In [27], Chung et al. designed an outdoor navigation system that combines augmented reality technology and GPS localization. By using user GPS coordinates, the system renders AR guidance arrows to reduce errors and improve overall exploration experiences. This design choice aimed to provide users with intuitive and real-time visual cues, reducing errors in the navigation process.

In [28], Sasaki et al. introduced a novel system designed to enhance the tourist experience through a fusion of Augmented Reality (AR) based on location and object-recognition AR. Their system aimed to provide comprehensive guidance and information about sightseeing spots, utilizing intuitive pictograms to facilitate tourism activities. This innovative approach signifies a valuable contribution to the field, as it not only integrates technological elements but also caters to practical, real-world scenarios in the domain of tourism.

In [29], Maia et al. proposed a location-based AR named *LAGARTO*, an authoring tool for location-based games that support AR features. This tool was developed to allow users with no programming skills to create single-player and multiplayer location-based AR games by using graphical notations. Unfortunately, the current implementation of the above-mentioned systems is based on traditional sensor fusion and a GPS localization service; the AR anchor point position accuracy depends on the quality of the device sensors.

### 2.2. *VSLAM* Implementation Methods

In order to address the limitations of conventional smartphone sensor fusion methods using the GPS and compasses in the *LAR* implementation, many studies have harnessed the potential of *VSLAM* to empower the system to simultaneously map the environment and localize the user’s device within it.

In [30], Tourani et al. offered a comprehensive overview of the current *VSLAM* approach and technology. This study synthesizes insights from fifty impactful articles published in the domain. This extensive review not only aggregates existing knowledge but also provides an in-depth analysis of the theoretical foundations that have shaped the field of *VSLAM*. Moreover, the depth of the literature survey contributes to the identification of trends, gaps, and emerging directions in *VSLAM* research.

In [31], Mur-Artal et al. introduced *ORB-SLAM2*, a complete Simultaneous Localization and Mapping (SLAM) system for monocular, stereo, and *RGB-D* cameras, including map reuse, loop closing, and re-localization capabilities. The system works in real-time on a standard central processing unit in a wide variety of environments from small hand-held indoor sequences to drones flying in industrial environments and cars. It enhances the trajectory estimation with metric scales, contributing to a lightweight localization for unmapped regions.

In [32], Xu et al. improved an *ORB RGB-D SLAM* with a 2D *Occupancy Grid Map (OGM)*. The 2D OGM is built with the 3D camera poses estimated by *Visual SLAM (vSLAM)* and laser scans extracted from the point cloud observed by the camera from those poses. In addition, *Robot Operating System (ROS)* visualization tools are used to overlay real-time current camera poses and observations by virtual laser scans on the OGM. The evaluation results showed its high localization accuracy. Unfortunately, this approach can only be implemented in indoor scenarios.

In [33], Kiss et al. proposed an improvement of *ORB-SLAM* using the GPS and IMU data of the images to make it more robust for datasets with low frame rates. This work was motivated by a scarce dataset where *ORB-SLAM* often loses track because of the lack of continuity. This approach is basically a combination of three data inputs from the SLAM world coordinate system, the SLAM camera coordinate system, and the GPS coordinate system. The authors used GPS data for the SLAM tracking part in which the position for the next frame is predicted. The corresponding GPS data were read for every image dataset. The method in this study is similar to our proposed approach, which also uses GPS and location data to improve the localization accuracy of the *VSLAM*. However, this study needs to capture large outdoor image references with its GPS and *Inertial Measurement Unit (IMU)* manually using an *unmanned aerial vehicle (UAV)*.

In [34], Sukimura et al. introduced an open-source visual *SLAM* framework with high usability and extensibility to enhance spatial understanding and AR experiences in the context of *VSLAM*. By addressing the limitation of *ORB-SLAM2*, their work contributes to the practical deployment of *VSLAM* solutions in a monocular camera device, such as smartphones. We adopted this work as a basic *VSLAM* library in this paper.

In [35], Jinyu et al. used visual sensors, such as monocular or multiple cameras, to estimate the camera pose and the scene structure according to multi-view geometry theory. The *VSLAM* algorithm works by extracting features from the camera images and using them to estimate the camera’s motion and the 3D structure of the scene. The algorithm typically consists of two main components: feature extraction and tracking, and pose estimation and mapping. This study gives a detailed explanation of the *VSLAM* implementation in monocular cameras augmented reality (AR), including the basic algorithm and limitations.

In [36], Manni et al. proposed a system to address the *VSLAM* limitation, which requires the scanning of the environment and tends to suffer from low resolutions and noises. Because of them, the system cannot predict the actual position and scale of the object due to the scale ambiguity. This study overcomes the limitations by allowing the user to capture a single view of the object using an Android smartphone equipped with a single RGB camera and supported by *Google ARCore*. However, their method typically needs the user input of an initial pose estimation for them to start working correctly. This is inconvenient and often infeasible when users need to capture a large number of image references.

### 2.3. Vision-Based Tracking Methods

To improve the AR anchor point precision in outdoor scenarios, many studies have proposed image-based tracking inside the *LAR* system.

In [37], Santos et al. designed an *LAR* application called *AzulejAR*, which combines sensors, the GPS, and vision-based tracking to improve the registration in mobile AR applications. The system uses offline image references to improve localization accuracy. Moreover, the system relies only on the inertial sensors to improve its accuracy and does not use the advantage of the *SLAM* algorithm.

In [38], Zhou et al. developed a tourist attraction guide system combining image-recognition technology and AR technology. This approach is similar to our method of increasing the anchor point accuracy by involving the image reference database. However, the system was developed in Unity and needs more resources than the native applications. Our previous study reveals that the native *Android* application has a more efficient computation resource allocation compared to the *Unity* implementation in outdoor *LAR* scenarios. Additionally, in the implementation, a vast number of image references needed to be manually captured and stored in the dedicated database.

## 3. Previous Work

In this section, we briefly introduce the previous work related to this study in outdoor and indoor scenarios.

### 3.1. Outdoor Navigation System Implementation

In [39], we designed and implemented an outdoor *LAR* solution for a bus route planning system. It has the feature of supporting the action of a bus user in an innovative way by putting an additional AR information layer on the smartphone camera screen and giving the instruction assistant that leads the user a way to the nearest bus stop. It also offers real-time bus information from official government data. This solution was highly appreciated by user acceptance in terms of overall navigation experiences [40]. Unfortunately, the implementation of this system does not leverage the *SLAM* and visual tracking approach, where the AR anchor point position accuracy depends on the quality of device sensors, making the navigation experience different in each device.

In [41], we conducted the preliminary implementation of the *ARCore* engine with the sensor fusion approach both in *Android* native and *Unity* for the outdoor navigation system. This *LAR* application integrates the sensor fusion (GPS and compasses) with the *VSLAM* technique. The result showed that *Android* native offers a better resource computation than the *Unity* platform. Thus, in this study, we leverage the implementation with *VSLAM* and *GSV* to support the outdoor scenario in the *Android* native environment.

### 3.2. Indoor Navigation System Implementation

In [42], we designed and implemented an indoor navigation system by employing the *VSLAM* technique in a *LAR* system that focuses on indoor localization for the ambulation system. This *LAR* system uses augmented reality (AR) on a smartphone to display the moving direction arrow on the camera view. After initializing the current position via the *QR* code, it uses a smartphone’s camera and gyroscope to follow the user’s movements inside a building using the *SLAM* approach. Unfortunately, the proposed navigation system is not suitable for outdoor scenarios due to the necessity of many QR codes as markers for navigation accuracy improvements.

## 4. Proposal of Anchor Precision Enhancement Method

In this section, we present the anchor precision enhancement method by integrating *VSLAM* and *Google Street View* for the *LAR* application. The method consists of two phases: the prototype development phase and the evaluation phase. The prototype development phase is the one for building the *LAR* application, which utilizes the *VSLAM* and cloud-based matching from *Google Street View*. In the evaluation phase, the prototype will be compared with a common GPS-based *LAR* application. The result was then measured and analyzed with predetermined research variables, such as distance error and running load metrics.

### 4.1. Insight of Proposal

The critical role of *Google Street View* in this proposal is underpinned by the study of Biljecki et al. [43]. They showcased that the extensive reference of geospatial and visual data offered by *Google Street View* can be useful for location-based service applications. We rely on the cloud-based matching module that leverages the extensive visual reference database of *Google Street View*. This extensive database not only enhances the visual reference matching process but also provides accurate geospatial data for improved localizations without collecting reference image data manually [44]. Building on these insights, our study aims to create a more sophisticated *LAR* system that leverages *VSLAM* and the rich visual reference data from *Google Street View* to improve position accuracy in outdoor environments.

### 4.2. Three Parameters for Localization

**Figure 2:**
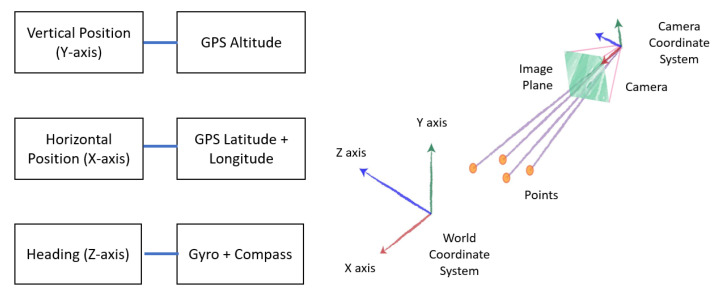
Mapping concept of proposed localization.

Generally, *LAR* systems work by using a combination of sensor data, geospatial information, computer vision, and advanced algorithms to create an augmented reality that is seamlessly integrated with the user’s physical surroundings, providing immersive and contextually relevant experiences. The outdoor position and localization depend on three parameters, which are the horizontal position (X-axis), the vertical position (Y-axis), and the heading of the user’s device (Z-axis) [45,46]. These axes are fundamental for defining the position, orientation, and movement of objects within an augmented reality environment. Figure 2 depicts the mapping concept and the sensors that are being used to obtain each position in the proposed localization method.

The horizontal position refers to the device’s latitude and longitude coordinates on the Earth’s surface. The vertical position refers to the height or the elevation of the device above the reference point, typically the ground level. The heading refers to the orientation or direction in which the device is pointing. In the context of *LAR*, the transformation between the camera coordinate system (user’s device) and the world coordinate system (physical environment) is a crucial part of ensuring that augmented objects are correctly situated in the user’s real-world view. Our approach intends to enhance the accuracy of the three axes’ position with *VSLAM* and the *Google Street View* imagery database.

### 4.3. Structural Design of Proposed System

**Figure 3:**
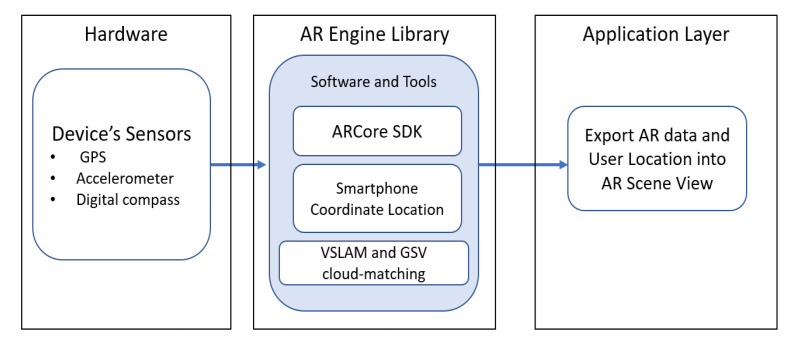
Structural design of the proposed system.

In this study, the proposed LAR system utilizes a combination of hardware and software components to provide users with contextually relevant virtual content aligned with their real-world surroundings. Figure 3 shows the structural design of the proposed *LAR* application.

#### 4.3.1. Input

The main input of the proposed system includes the sensors embedded within the user’s device. They typically include the GPS, accelerometers, and digital compasses. The GPS sensor is vital for geospatial positioning, allowing the system to precisely determine the user’s location on the Earth’s surface. Accelerometers measure acceleration forces, assisting in motion sensing and understanding the user’s movement. Digital compasses provide orientation data, ensuring the AR content is correctly aligned with the user’s physical surroundings.

#### 4.3.2. Process

The AR engine tool serves as the core computation module behind the location-based AR application system. This tool consists of software libraries, frameworks, and development kits. In this paper, we use *ARCore SDK for Android version 1.39*. The SDK provides the necessary *application programming interfaces (APIs)* to access and process sensor data, map the user’s environment, and render 2D or 3D objects in real-time. The AR engine is responsible for sensor fusion services that combine data from various sensors for accurate tracking and positioning. After obtaining the user location coordinates (latitudes and longitudes), the proposed system enhances the sensor data output with an additional *VSLAM* module and then sends the location-related data to *Google API* for the cloud-matching process from the *Google Street View* database.

#### 4.3.3. Output

Finally, based on the user location data, the proposed system generates the AR anchor objects. The *LAR* application accesses sensor data, communicates with the AR engine, and determines how and where the AR objects are placed within the user’s field of view to create immersive and contextually relevant experiences.

### 4.4. System Architecture

The proposed *LAR* system integrates cloud-based services into the smartphone technology to provide the *LAR* functionality. The components of the proposed system include built-in smartphone sensors, the *Google ARCore SDK for AR rendering*, the *VSLAM* module for localization, and cloud-based services that facilitate the retrieval and processing of geospatial data from *Google* services, including *Google Maps API*, *Google Earth*, and *Google Street View*. The AR object-related data, such as the anchor ID and POI information, are stored in the dedicated server. All the external service communications use the *JavaScript Object Notation (JSON)* format.

**Figure 4:**
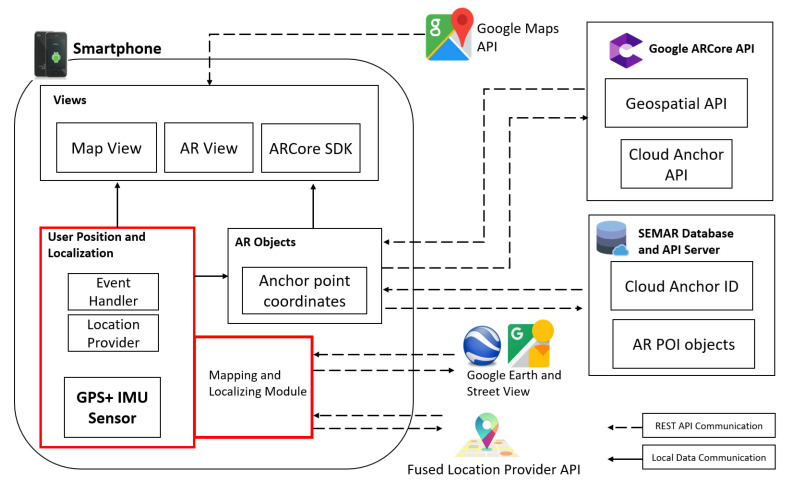
Proposed system architecture.

Our approach heavily relies on data from smartphone sensors, particularly the *GPS*, and *ARCore SDK for AR object rendering*. It begins with obtaining raw user location through *GPS* and *IMU* sensor data; the accuracy of raw coordinate position data is enhanced by the *Fused Location Provider API* and processed by the *Location Provider* module. The *user position and localization* module employs *VSLAM* technology to map the environment and localize the user within it accurately. *VSLAM* can refine the user localization, especially for altitude (vertical position), while *Google Earth* and *Google Street View* enrich the reference of geospatial data and visual image references. The AR environment is developed by the utilization of *Google ARCore*, which offers the generation of AR objects into real-world coordinates. AR objects are virtual elements placed within the *AR View*, representing POIs or other contextual information. User interactions are managed by an event handler to display the AR object and *Map View*. Then, the *SEMAR* server [47] is used to store the AR content persistence data. The persistence data include the anchor ID, latitude, longitude, altitude, and heading values of the generated AR objects. The *SEMAR* server offers big data environments with built-in functions for data aggregations, synchronizations, and classifications with machine learning, and plug-in functions that access the data through the *Representational State Transfer (REST) API* in the *JSON* format. Figure 4 depicts the detailed system architecture of the proposed *LAR*.

### 4.5. *VSLAM* and Cloud-Based Matching Implementation

In [35], Jinyu et al. showed that *Visual SLAM (VSLAM)* is a technique that uses visual sensors, such as monocular or multiple cameras, to estimate the camera pose and the scene structure according to the multi-view geometry theory. The *VSLAM* algorithm typically consists of two main components: feature extraction and tracking, and pose estimation and mapping.

The feature extraction and tracking stage involves identifying distinctive features in the camera images and tracking them across multiple frames. The projection of the 3D point $X_{j}$ to image *i* can be represented as

(1) $$x_{ij}=h\left(C_{i},X_{j}\right)=\pi \left(KR_{i}^{T}\left(X_{j}-p_{i}\right)\right).$$

In this equation, the 3D world point is represented as $X_{j}$ and the camera pose as $C_{i}=\left(R_{i},p_{i}\right)$, where $R_{i}$ is the rotation matrix and $p_{i}$ is the camera position of image *i*. *K* is the camera intrinsic matrix, and $\pi \left(x,y,z\right)={\left(x/z,y/z\right)}^{T}$ is the projection function.

In the pose estimation and mapping stage, the algorithm uses the tracked features and the camera’s motion to estimate the camera’s pose and the 3D structure of the scene. This involves solving a system of equations that relates the 2D image coordinates of the tracked features to their 3D positions in the scene. The resulting pose and map estimates are then refined using techniques such as bundle adjustment, which optimizes the estimates to minimize the re-projection error.

**Figure 5:**
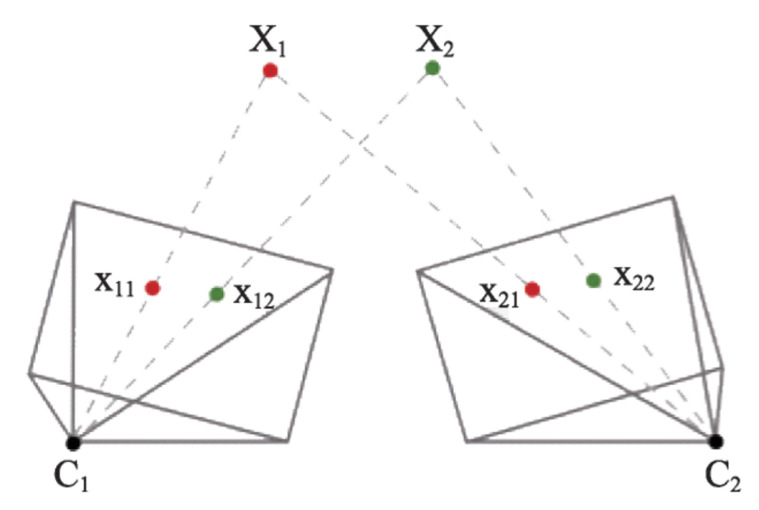
Illustration of multiple view geometry [35].

The *VSLAM* algorithm aims to estimate the camera poses $C_{i}$ and the 3D points $X_{j}$ that best explain the observed image data, while also considering the uncertainty associated with the measurements and the camera motion. Figure 5 illustrates the geometric relationship between the 3D world point $X_{j}$ and its projection onto the 2D image plane of camera *i*. It visually depicts how the camera’s intrinsic parameters, rotation, and translation affect the projection of the 3D point onto the 2D image plane, providing a visual representation of Equation (1).

In this paper, the *OpenVSLAM* is leveraged as a *VSLAM* library. This library is a monocular modality framework of the visual SLAM introduced by Sumikura [34]. *OpenVSLAM* was selected based on its robustness and compatibility with various camera models, including monocular smartphone cameras. The detailed *OpenVSLAM* algorithm and implementation code can be accessed in [48]. *OpenVSLAM* is basically a derivative version of *ORB–SLAM*, *ProSLAM*, and *ORBSLAM2*, which can potentially overcome some of the limitations of *ORB-SLAM2* [31,49,50]. Unlike other visual SLAM techniques, *OpenVSLAM* can store and load map databases for further localizations.

One of *VSLAM*’s limitations is that it can suffer from drift over time, which can lead to errors in the estimated camera pose and 3D scene structure. This drift can occur due to errors in the feature tracking, the camera calibration, or the accumulation of small errors in the estimated camera motion over time. Additionally, *VSLAM* can struggle in environments with low texture or repetitive patterns, as it may be difficult to identify distinctive features for tracking. Thus, GSV cloud matching is utilized to provide image tracking and pose refinement.

**Figure 6:**
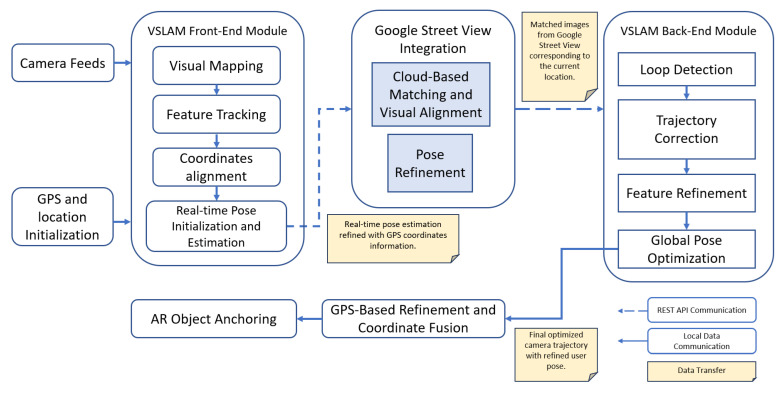
Block diagram of proposed localization method.

For the cloud matching process, *Google’s Geospatial API* was used as an automation computation cloud service. This API service is based on trillions of point cloud imageries from the *GSV* API service [51]. This imagery database can support *VSLAM* in the localization and mapping process. This database allows the system to recognize and match features in the current scene with those in the database. It retrieves accurate positioning by comparing the device’s camera view with the GSV huge database, after initial filtering based on the user’s approximate geolocation. The step-by-step explanation of how *VSLAM* can work in conjunction with *Google Street View* can be seen in Figure 6.

The LAR system initiates with the provided GPS coordinates. The GPS coordinates serve as initial estimates for *VSLAM*. In the *Visual Mapping* phase, the *VSLAM* module starts by capturing video frames using the device’s camera and extracting visual features such as key points and descriptors from each frame. The front end of a *VSLAM* system is responsible for the initial stages of processing, particularly the real-time aspects of feature extraction, feature matching, and pose estimation. These visual features are utilized to construct a visual map of the environment. In the front end of *VSLAM*, the algorithm identifies distinctive features in the camera’s field of view, often referred to as *key points* that could be corners, edges, or other unique visual elements. Once features are extracted in successive frames, the algorithm matches these features between frames. This matching process establishes correspondences, allowing the algorithm to track the movement of these features over time.

The *VSLAM* module determines the device’s initial position and orientation to initialize its pose estimation. An initial estimate of the camera’s pose is obtained from previous frames. While *VSLAM* primarily relies on visual information for real-time pose estimation, the initial GPS coordinates are used to refine or initialize the camera’s pose. This integration helps align the visual information obtained through *VSLAM* with the global coordinate system provided by the GPS.

In aligning with *Google Street View (GSV)*, the GPS coordinates are typically included in the query to *Google Street View* along with the visual features. This information helps *GSV* retrieve relevant images corresponding to the device’s location. The GPS coordinates act as a key parameter for the cloud-based matching process. Each *GSV* image is associated with its own *GPS* coordinates and heading information. This image data consists of high-resolution panoramic images captured at specific locations. *VSLAM* compares the current camera view with the *GSV* images to find visual correspondences. By matching the visual features extracted from the camera frames with those in the *GSV* images, the module can estimate the camera’s position and orientation relative to the *GSV* data.

After obtaining the feature matching result, *Loop Detection and Correction* are implemented. The *VSLAM* back-end module looks for the loop closure, which will occur when the device re-visits a previously visited location. GPS coordinates are also used to detect the re-visited locations. When a loop closure is detected, the system corrects any accumulated error in the estimated trajectory. This step is crucial for maintaining accurate localization over an extended period. The limitations of *vSLAM*, such as trajectory drift or scale drift, which are common in the case of monocular camera input, are resolved via a global optimization process [52].

Using the visual correspondences between the camera frames and *Street View* images, *VSLAM* refines the device’s pose estimation, aligning it more accurately with the *GSV* data. This refinement process helps improve the accuracy of the device’s position and orientation for pose refinements. Finally, with the refined GPS localization and pose estimation, *VSLAM* can localize the device within the mapped environment more accurately and the LAR system can use the user position to place an anchor point overlay of augmented reality content or virtual objects onto the real-world view.

### 4.6. Prototype Implementation

**Figure 7:**
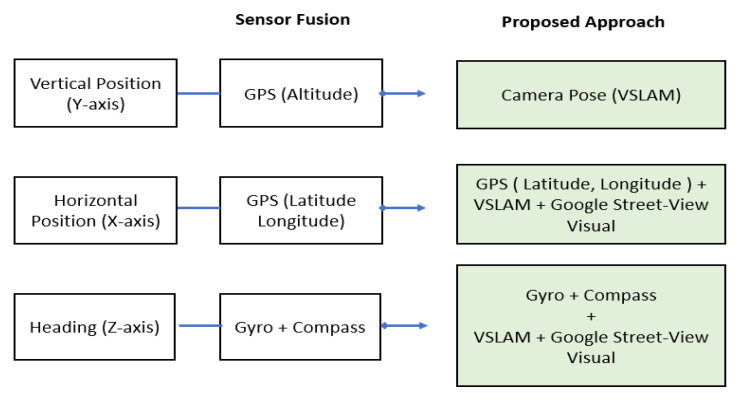
Overview of *LAR* method in this study.

This study develops the *LAR* system powered by *ARCore SDK* in the *Android* native environment. This prototype harnesses the advanced capabilities of *VSLAM* technology, working in synergy with the extensive visual reference database of *Google Street View*. *VSLAM* technology excels in offering precise and dynamic user localization, aligning the user’s position with accuracy. In this prototype, Google’s innovative cloud-based matching services were implemented to refine user localizations by leveraging machine learning and cloud-based computer vision from *Street View* visual reference imagery. This prototype reflects our proposed approach, where the main idea is to enhance the anchor point coordinates by aligning the captured camera frames that are enhanced with *VSLAM* localization with the known street-level views. Figure 7 depicts the proposed method components that have been used to determine the anchor point coordinates (position and axis) in comparison with common sensor fusion *LAR* solutions.

**Figure 8:**
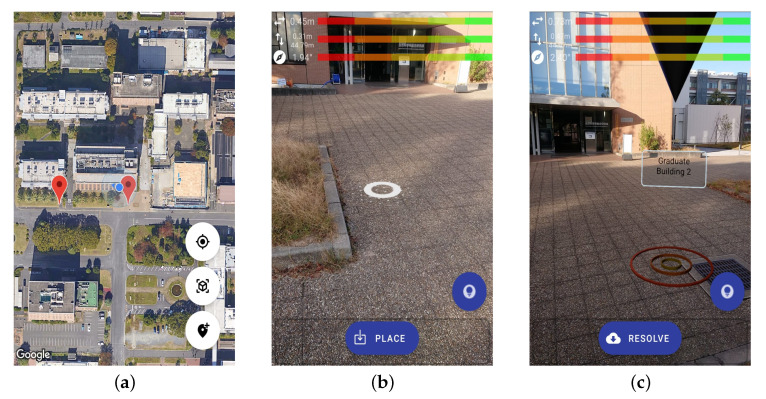
User interface samples of the proposed system. (**a**) 2D map interface. (**b**) *VSLAM* detecting surface for vertical position. (**c**) AR scene interface.

The main feature of the proposed system is to detect the user’s location and to show the stored AR object to the user. Figure 8 shows the interface samples of the *LAR* prototype. When the user opens this application, the prototype provides a 2D map as an object browser to the user. This interface likely allows the user to explore the surrounding area and potentially view the locations of stored augmented reality (AR) objects within the map (Figure 8a). The *VSLAM* technology is used to map the environment and localize the user position. In this context, a visual representation of how the system detects and processes vertical positioning data using the *VSLAM* technology is displayed with overlaid featured points on the image frames (Figure 8b). Since this prototype is in the preliminary stage, the AR anchor objects are not designed for navigation guidance. The prototype only facilitates the AR object generation for anchor point testing purposes, such as POI location (Figure 8c). Following the earlier discussion of the core *LAR* positioning variables, which are the horizontal position (X-axis), vertical position (Y-axis), and the heading of the user’s device (Z-axis), the prototype displays the values of these variables at the top of the screen to facilitate testing.

### 4.7. Comparison in Evaluation Phase

The proposed system shows high scalability and flexibility for outdoor *LAR* scenarios, as long as there is a clear line of sight and the *Google Street View (GSV)* service is available. In the evaluation phase, this prototype will be compared with the traditional sensor fusion to investigate the accuracy of AR anchor point location coordinates and the error distance between the designated anchor point coordinates and those estimated coordinates that are obtained using both the sensor fusion approach and the proposed approach. This comparative analysis will provide valuable insights into the effectiveness of each method in ensuring precise location-based AR experiences.

## 5. System Evaluations

In this section, we evaluate the feasibility of the proposal, compare the anchor point positioning accuracy with conventional GPS-based methods through experiments, and run the profiling test to measure the running load performances.

### 5.1. Testing Environment

The testing environment and setting for this evaluation were meticulously designed to evaluate the proposal for the *LAR* anchor precision enhancement. With the goal of addressing the limitations of traditional sensor fusion methods in AR implementation, our research focuses on outdoor AR scenarios where the accuracy of *LAR* applications is often challenged. The research conducted rigorous evaluations in diverse outdoor scenarios to verify the effectiveness of the proposed approach. It included 10 urban settings in Universitas Brawijaya, Indonesia, and Okayama University, Japan. The testing locations included specific university buildings, student dormitories, and common public areas such as parking areas. We also chose the POI locations based on the GSV services availability. The choice of diverse outdoor environments aimed to emulate real-world conditions where *LAR* applications are commonly used by people, especially students, such as pedestrian navigation, tourism, and outdoor gaming. In this testing, we did not quantitatively compare the GSV performances in different light conditions. The goal of this study was to analyze the feasibility of GSV integration in the LAR implementation to enhance the AR object anchoring accuracy; we mainly focused on the comparison between the two approaches of LAR technologies in good lightning conditions when sunlight was available.

### 5.2. Testing Two Prototypes

This study first compares the proposed approach with a common sensor fusion *LAR* solution to prove our proposed system’s accuracy and performance. Both prototypes were implemented as *Android* native applications that have the main features of basic location-based AR, such as rendering AR scenes, obtaining user fine and coarse locations using smartphone GPS and mobile data, and computing the user location data to provide location-based service to display the AR object based on user location. These prototypes commonly rely on user localization accuracy to generate AR objects. Each prototype represents a different approach to *LAR* anchor precision.

#### 5.2.1. First Prototype

The first prototype relies on traditional sensor fusion techniques for user localization. We have used *DroidAR SDK* [53] along with the *Android Studio* to develop the sensor fusion *LAR* application. *DroidAR* library provides an all-in-one library to access the user location and also render the AR object based on the user coordinates. The smartphone’s *GPS* sensor and mobile data play pivotal roles in determining the user’s location. The *GPS* sensor provides the fine location data, offering the user’s geospatial coordinates, whereas the mobile data contribute to the coarse location. The two data sources of the fine and coarse locations are processed to compute the user’s complete location. This prototype is the typical conventional sensor fusion method employed in many *LAR* applications.

#### 5.2.2. Second Prototype

The second prototype harnesses the *VSLAM* technology in conjunction with *Google Street View*. To avoid inconsistency in the performance data, the same *Android*-based smartphone is used in all experiments. Table 1 shows the specification of this *Android* device.

### 5.3. Testing Process

As a way to concretely embody a view of the AR anchor point or the POI position accuracy, a direct comparison was conducted between the traditional sensor fusion and our proposed approach. In our study, we initially established the true coordinates for 10 POIs by specifying their respective latitude and longitude values. Subsequently, we employed both *LAR* applications to create the AR object at each POI and collected the estimated coordinates. For each of the 10 Point of Interest (POI) locations serving as anchor points, we carefully set up the outdoor testing environment. This included ensuring good lighting conditions when the sunlight was always available, ensuring the GPS data and GSV service were available, and preparing the LAR system for data capture. At each anchor point, we performed three sequential measurements using both LAR systems using the same specification testing device. We captured the estimated location of the anchor point coordinates in both of the LAR prototypes and then sent this location information to the SEMAR server for further calculation and analysis. This sequential approach was crucial to capturing variations and estimation data consistency in each experiment at each POI location. We recorded both the estimated position coordinates provided by the LAR system and the ground truth data, which represented the true position coordinates. This process was repeated for 10 POI locations on different days so we obtained a total of 30 measurements (estimated anchor point location coordinates) for each LAR prototype.

**Figure 9:**
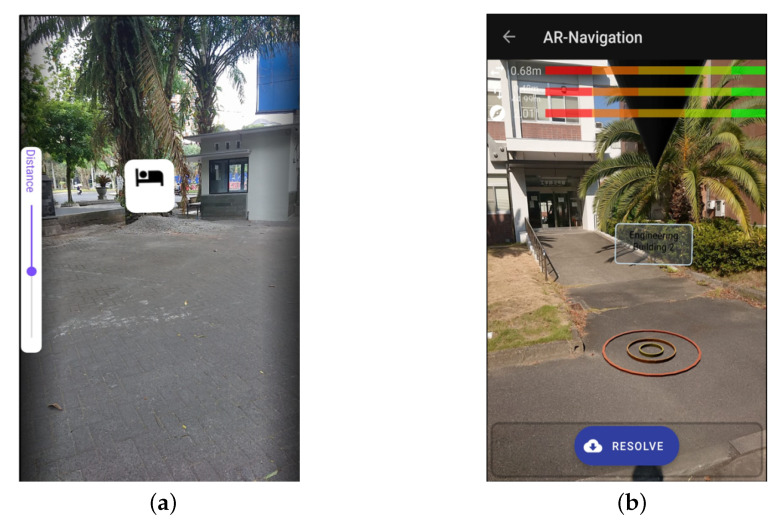
Screenshots of two *LAR* application interfaces in the experiment. (**a**) Sensor fusion *LAR* user interface. (**b**) Proposed *LAR* user interface.

We conducted two iterations of the experiments using the same smartphone to compare the performance of the traditional sensor fusion-based *LAR* and our proposal. In the first iteration, we utilized the traditional sensor fusion *LAR* application. Then, in the second iteration, we implemented our proposal. From them, we obtained the estimated anchor point coordinate and calculated the distance to the true anchor location as the estimation error. The true anchor location is the actual designated coordinate that we want to place the AR POI, where the estimated location coordinate is generated by the *LAR* application to display the POI. Figure 9a depicts the misalignment of common sensor fusion LAR developed using *DroidAR SDK*; the AR anchor point is glitching due to this method only relying on latitude, longitude, and a default zero value of altitude. On the other hand, the proposed LAR system can generate the AR anchor point steady on the user’s ground surface perspective by using feature tracking of *VSLAM* (Figure 9b).

### 5.4. Outdoor Localization Accuracy Comparison

To verify the effectiveness of the proposed *LAR* system, 10 samples of various AR anchor point locations were predefined for the accuracy evaluation. In this case, the proposed *LAR* application was compared with the traditional GPS and sensor fusion approach in terms of anchor point accuracy. Instead of relying on a single measurement, we conducted multiple accuracy measurements at each anchor point.

#### 5.4.1. Error Distance Calculation

In the evaluation, we aimed to evaluate the feasibility and accuracy of our proposed Location-Based Augmented Reality (LAR) system in outdoor environments. The evaluation specifically focused on assessing the error distance between the true location and estimation of anchor points at each Point of Interest (POI) location using *Mean Error (ME)*, *Standard Deviation (SD)*, and *Root Mean Square Error (RMSE)*. ME provides an overall measure of accuracy by calculating the average difference between the true and estimated distances. SD measures the spread or variability of the value differences, providing insight into the consistency of the estimation data. ME and SD were computed to compare the precision of the AR anchor positioning for each POI location. The composite RMSE evaluates overall accuracy by considering the distribution and value of distance errors. The estimated location coordinates (latitude and longitude) were generated by both prototypes on the server. The distance data between the true location coordinates and the estimated ones were calculated as the estimation errors with the *Haversine formula*.

The *Haversine formula* is the mathematical formula used to calculate the distance between two points on the surface of a sphere, given their latitudinal and longitudinal coordinates [54]. The detailed equation of the *Haversine formula* is described in Equation (2). In the context of the distance error testing, the *Haversine formula* is employed to compute the great-circle distance between the true location $\left(\phi_{A},\lambda_{A}\right)$ and the estimated location $\left(\phi_{B},\lambda_{B}\right)$ for each POI coordinate.

(2) $$\theta =2\arcsin\left(\sqrt{\sin^{2}\left(\frac{\Delta \phi }{2}\right)+\cos\left(\phi_{A}\right)\cos\left(\phi_{B}\right)\sin^{2}\left(\frac{\Delta \lambda }{2}\right)}\right),$$

where:

$$\begin{array}{cc} A & =\mathrm{true}\;\mathrm{coordinate};\\ B & =\mathrm{estimated}\;\mathrm{coordinate};\\ \theta & =\mathrm{central}\;\mathrm{angle}\;\mathrm{between}\;\mathrm{two}\;\mathrm{points};\\ \phi_{A},\phi_{B} & =\mathrm{latitudes}\;\mathrm{of}\;\mathrm{two}\;\mathrm{points};\\ \lambda_{A},\lambda_{B} & =\mathrm{longitudes}\;\mathrm{of}\;\mathrm{two}\;\mathrm{points};\\ \Delta \phi & =\phi_{B}-\phi_{A}\;\left(\mathrm{difference}\;\mathrm{in}\;\mathrm{latitude}\;\mathrm{between}\;\mathrm{the}\;\mathrm{two}\;\mathrm{points}\right);\\ \Delta \lambda & =\lambda_{B}-\lambda_{A}\;\left(\mathrm{difference}\;\mathrm{in}\;\mathrm{longitude}\;\mathrm{between}\;\mathrm{the}\;\mathrm{two}\;\mathrm{points}\right). \end{array}$$

The distance *D* between two points is given by

(3) D=θ×6371km,

where:

$$\begin{array}{cc} D & =\mathrm{distance}\;\mathrm{between}\;\mathrm{the}\;\mathrm{two}\;\mathrm{points};\\ 6371 & =\mathrm{mean}\;\mathrm{radius}\;\mathrm{of}\;\mathrm{the}\;\mathrm{Earth}\;\mathrm{in}\;\mathrm{kilometers}. \end{array}$$

From the collected coordinate values, we calculated the value of the *Mean Error (ME)* and *Standard Deviation (SD)* for each POI location using Equations (4) and (5), consecutively. In order to provide an overall assessment of both methods’ performance across all locations, the overall ME and *RMSE* were also calculated on all measurement results with Equation (4) and Equation (6) [55].

(4) $$ME=\frac{1}{n}\sum_{i=1}^{n}\left|True_{i}-Estimated_{i}\right|,$$

(5) $$SD=\sqrt{\frac{\sum_{i=1}^{n}(|True_{i}-Estimated_{i}{\left|-ME\right)}^{2}}{n}},$$

(6) $$RMSE=\sqrt{\frac{1}{n}\sum_{i=1}^{n}{\left(True_{i}-Estimated_{i}\right)}^{2}},$$

where $True_{i}$ and $Estimated_{i}$ represent the true location coordinate and the estimated coordinate of the ith anchor point, respectively.

#### 5.4.2. Error Distance Comparison Result

Here, *ME*, *SD*, and *RMSE* were used to find whether a significant difference in the estimation accuracy exists between the proposed method and the conventional sensor fusion *LAR* method for outdoor scenarios. Table 2 presents the results of the location estimation error comparison for five *Points of Interest (POIs)* inside the campus area of Universitas Brawijaya, Indonesia. Each POI is associated with its true coordinates (latitude, longitude) and the estimated coordinates from both the conventional sensor fusion and proposed *Location-Based Augmented Reality (LAR)* approaches. The estimation error, expressed in meters, represents the distance between the true and estimated locations for each POI. The *ME* indicates the average estimation error across all POIs, offering insights into the overall precision of each LAR method. In this context, a lower *ME* signifies a closer alignment of estimated positions with true positions. The *SD* complements the ME by quantifying the variability or spread of estimation errors. A smaller *SD* indicates more consistent and reliable estimations. Additionally, the *RMSE* is provided as a comprehensive metric, considering both the magnitude and variability of errors. The *RMSE* is particularly useful for assessing the overall accuracy of the LAR methods, with a lower value indicating better performance.

**Figure 10:**
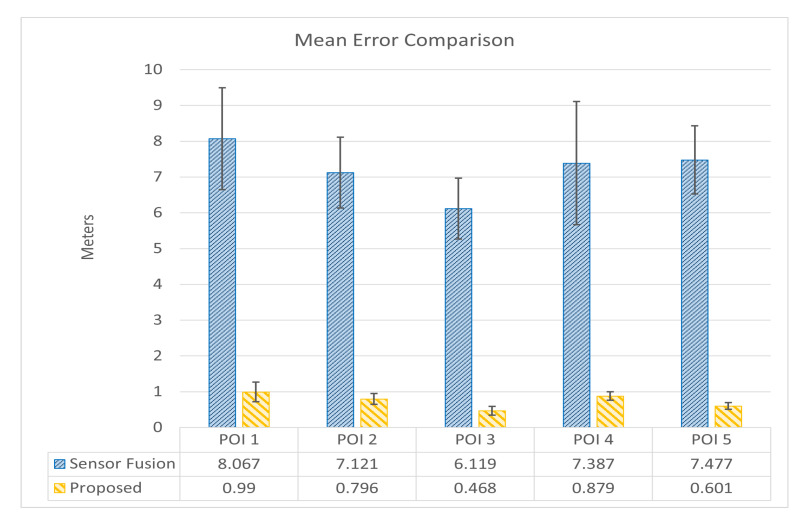
Anchor point distance errors comparison of *LAR* prototypes for Universitas Brawijaya.

The experiment results can be comprehended more by the visual representation of the data in Figure 10. All the POIs are comprehensively summarized by the *overall ME* and *RMSE* values. The X-axis corresponds to the individual POIs, while the Y-axis depicts the estimation errors in meters. This graphical representation allows for a quick comparison of the magnitude and distribution of errors across different locations. Both approaches have a consistent pattern of errors across POIs in the estimation method. The result offers insights into both the average accuracy and the consistency of performances across different Points of Interest. The proposed LAR approach has a satisfactory level of accuracy, as proven by the overall ME of 0.747 m, which shows a smaller average difference between the estimated and true positions rather than the conventional sensor fusion method (ME = 7.234 m). Similarly, the proposed approach’s accuracy and consistency are confirmed by the overall RMSE of 0.770 m, while in the conventional sensor fusion, it is 7.262 m.

**Figure 11:**
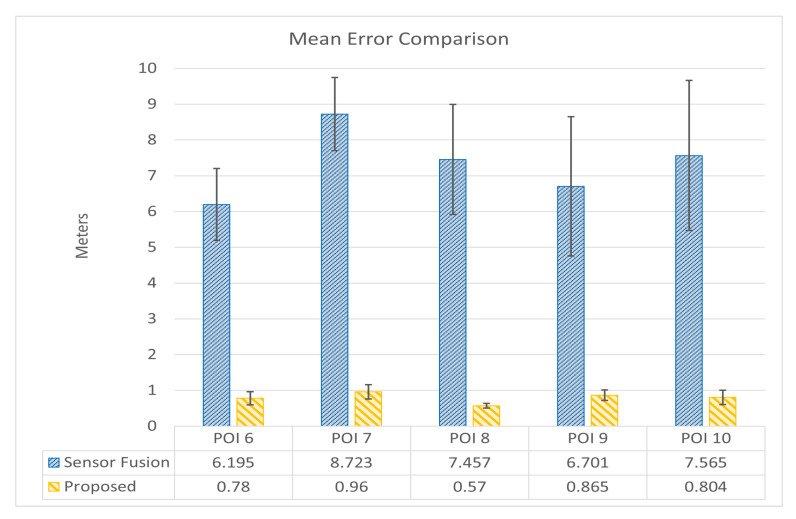
Anchor point distance errors comparison of *LAR* prototypes for Okayama University.

Figure 11 illustrates the location estimation error comparison for Okayama University, Japan. This figure is similar to the one for Universitas Brawijaya. It provides a comparison summary of the *ME* and *SD* of the estimation errors for each Point of Interest (POI).

Table 3 outlines the location estimation error comparison for the Okayama University campus between the conventional sensor fusion and the proposed Location-Based Augmented Reality (LAR) methods. Each *Point of Interest (POI)* presents true and estimated coordinates, estimation errors, and key statistical measures. For example, at POI 6, the proposed method achieves a remarkable Mean Error (ME) of 0.761 m and a low Standard Deviation (SD) of 0.183 m, indicating a consistent and precise estimation. This trend continues across POI 7, POI 8, POI 9, and POI 10, where the proposed LAR method consistently outperforms the conventional approach, and smaller ME values indicate better accuracy. The overall ME of 0.796 m and Root Mean Square Error (RMSE) of 0.806 m underscore the effectiveness of the proposed LAR method in enhancing location estimation accuracy across diverse Points of Interest within the study area.

When compared with the conventional sensor fusion approach that only relies on GPS data and compass-related sensors, the advantage of the proposed method is to harness the GPS and the IMU-related data with the *VSLAM* mapping and *Google Street View* data as references, which generates the outdoor POI accurately. Thus, *ARCore* can seamlessly be integrated with *Google Street View* data and achieve better AR experiences. The proposal shows great accuracy and flexibility for outdoor anchor point scenarios when the area is covered by *Google Street View* reference imagery.

The experiment results confirm that the combination of *VSLAM* and *Google Street View* database references has great potential to enhance outdoor *LAR* application experiences, making them more accurate, engaging, and reliable.

### 5.5. Running Load Performance Testing

In order to conduct a further comparison with the conventional sensor fusion-based *LAR* application, we conducted experiments for measuring running loads by each *LAR* prototype application. They were obtained using *Android Studio Profiler*, a tool that can record the CPU usage in percentage and the memory usage in megabytes (MB) [56]. We ran AR features in both LAR prototypes for approximately one minute to measure the CPU load and the memory usage. The testing device was the same for both prototypes.

#### 5.5.1. CPU Usage Comparison

**Figure 12:**
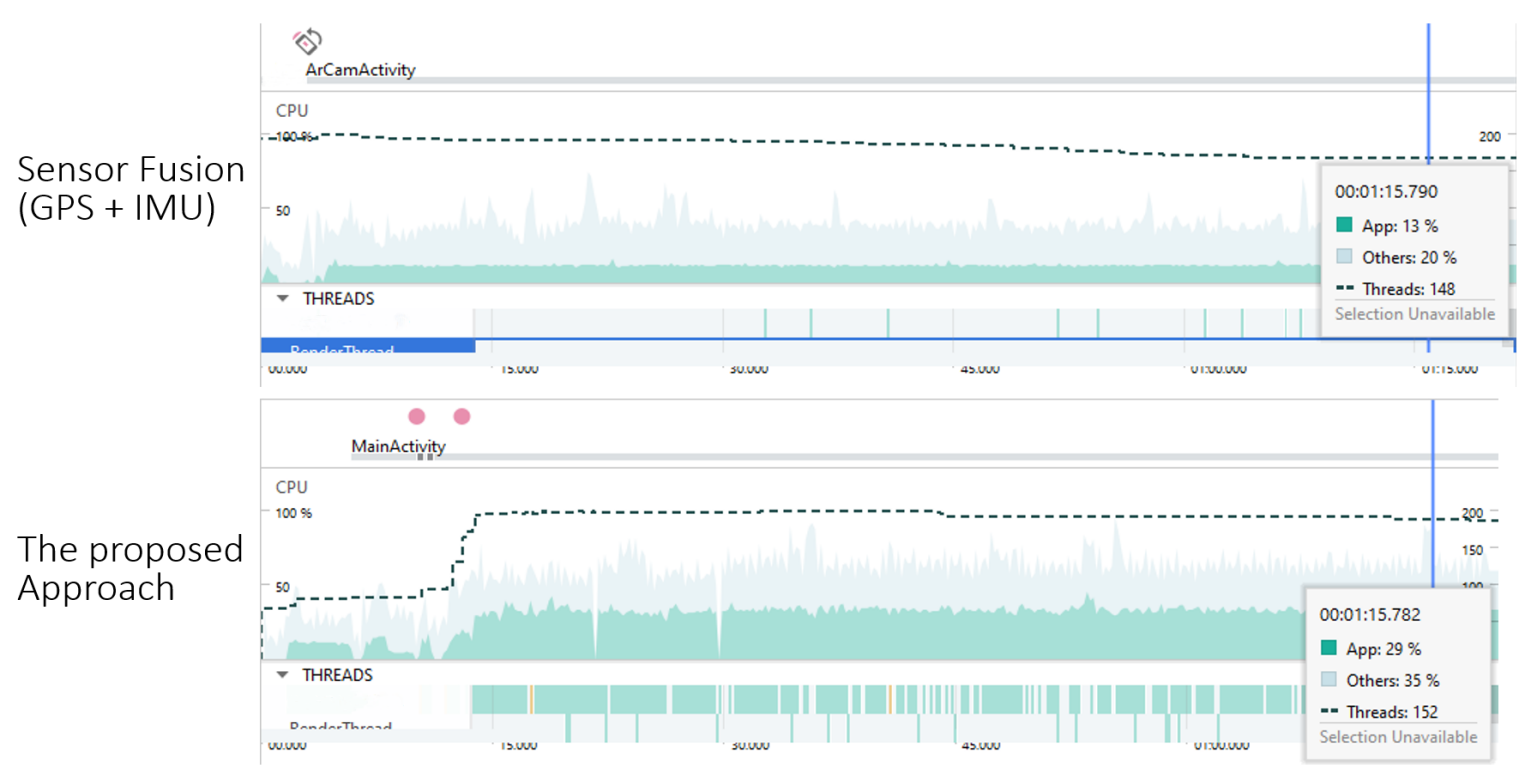
Comparison of CPU loads between proposal and conventional.

The CPU plays a pivotal role in handling the computational demands of real-time applications such as *LAR*. The CPU usage provides insights into the efficiency and resource requirements of the system. Figure 12 shows the CPU profiling results of both prototypes.

The proposed method shows an average CPU usage of 29 %. The conventional method uses an average CPU usage of 13%. This result shows that the proposal exerts a higher load on the device’s CPU compared to the conventional method. The increased CPU usage in the proposal can be implied from the more active threads to control *VSLAM* and cloud-based matching computation. *VSLAM* is the image-based technique that involves real-time camera pose estimation by tracking visual features across consecutive frames. *VSLAM* involves complex image processing and feature matching, which can be more computationally intensive than traditional sensor fusion methods relying on GPS and compass data.

Moreover, the integration of the cloud-based method may contribute to higher CPU utilization. The cloud-based system typically involves data retrieval, communication with remote servers, and real-time data processing, which further intensifies the computational loads. In the case of the proposal, leveraging cloud-based matching techniques introduces benefits such as enhanced access to extensive visual references from *Google Street View*. However, it also increases the processing complexity.

#### 5.5.2. Memory Usage Comparison

**Figure 13:**
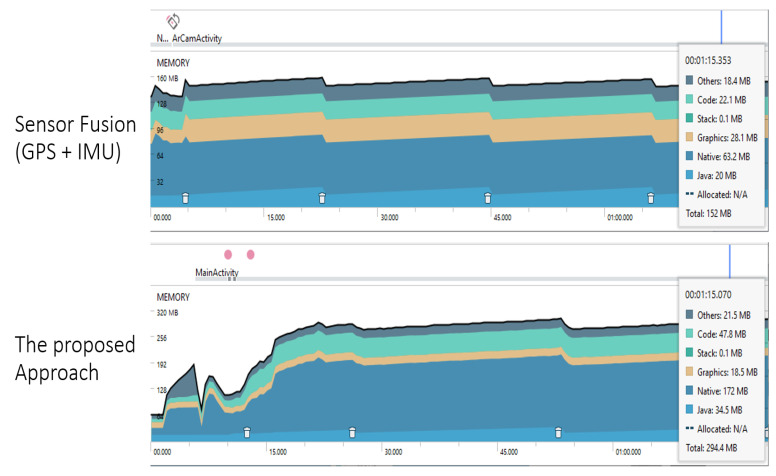
Comparison of memory usage between two approaches.

In terms of memory utilization, the proposed method consumes 294 MB of memory, whereas the conventional method uses only 152 MB. This indicates that the proposed method has a higher memory footprint compared to the conventional approach. The increased memory usage in the proposal can be linked to the processing of visual data from *Google Street View*. Storing and manipulating visual reference data from the cloud can require additional memory resources. It is important to note that while the proposal has higher memory usage, it may offer advantages in terms of improved accuracy and performance due to its reliance on visual references for precise positioning. The memory utilization of both approaches is depicted in Figure 13.

Overall, the proposed *LAR* method places higher computational and memory loads on the device compared to the conventional method. However, this increased load is accompanied by the potential for enhanced accuracy and alignment of the augmented reality content with the real world. Users will experience more precise location-based AR experiences at the cost of increased resource utilization. The choice between the two methods should consider the trade-off between the accuracy and the resource efficiency based on the specific application’s requirements and the capabilities of the target device.

#### 5.5.3. Discussion

Measuring anchor point precision and computation performance are important in studying the feasible approach to provide a good AR experience in the LAR solution because of the relatively higher contribution of location accuracy in the overall LAR user experience [57,58]. An interesting finding in the evaluation results highlighted the trade-off that comes with the proposed LAR implementation, which offers substantial accuracy enhancement and more precise alignment of augmented reality content with the real world, despite producing a greater computational and memory load on the device when compared to the conventional sensor fusion method.

In the context of Location-Based Augmented Reality experiences, the AR anchor point’s precise alignment can greatly affect user immersion and interaction; this trade-off is essential to be considered in the implementation practice. One key contributor to the increased computational load is the utilization of *VSLAM* and *GSV* data. *VSLAM* is a resource-intensive method in part because of its real-time camera posture estimate, which requires extensive image processing and feature matching. Furthermore, the incorporation of cloud-based approaches, particularly when utilizing GSV data, increases the computing load because of the retrieval of data, communication with external servers, and real-time processing.

However, the proposed approach has several benefits. When integrated with numerous images from Google Street View, *VSLAM*’s real-time camera pose estimation greatly improves the overall location accuracy. These technologies contribute to the precise and robust alignment of AR content in outdoor scenarios, as long as the GSV services are available. This indicates that the proposed method offers a compelling solution for LAR applications where accuracy is essential, like pedestrian navigation.

Since the research in this paper is at the preliminary stage, we have implemented the proposed approach in native Android based on our previous work insight. This native implementation makes fine-tuning applications possible to leverage specific features and capabilities of the Android platform. In AR, where real-time performance is crucial for a seamless user experience, optimizing for the native environment can result in better performance compared to cross-platform solutions.

So far, we have not quantitatively compared *OpenVSLAM* with other SLAM algorithms. We chose *OpenVSLAM* based on the comparison results in other papers, which show that it can be performed similarly to a very popular visual SLAM algorithm, ORB-SLAM, and it even outperformed it in terms of trajectory error for several datasets.

Considering the limitations of the GSV service’s availability and performance, experimenting with GSV cloud-matching performance in different lighting conditions will be our next topic to investigate. Ideally, when the GSV service is not available, both accuracy and data loss prevention are equally important. Therefore, it could be beneficial to fuse information from IMUs and SLAM in a hybrid or an adaptive sense.

Furthermore, the capabilities of the target device and the particular application requirements in specific scenarios will be interesting topics to explore. The proposed approach, which relies on *VSLAM* and GSV, is suitable for LAR implementation when high precision is crucial, even if it has relatively higher processing requirements.

## 6. Conclusions

This paper proposed the innovative approach of combining *VSLAM* and *GSV* to enhance the precision of *LAR* anchors in outdoor environments. The use of *GSV* in conjunction with *VSLAM* contributes to improved sensor fusion performance. By fusing visual data from *GSV* with other sensor inputs, the proposed approach can enhance the precision of LAR anchor points, addressing the accuracy limitations of traditional GPS and compass-based methods. The evaluations using 10 public locations with multiple experiments show that the proposed method achieves higher accuracy than the conventional sensor fusion method. At the same time, the proposal needs more computation resources than the conventional method. This approach can be implemented where the *GSV* service is available in a certain location. In future studies, we will investigate the feasibility of the proposed approach in different lighting conditions, extend the implementation of the proposed approach for outdoor navigation scenario implementation, and continue to evaluate the performance of the overall navigation experience.

## Figures and Tables

**Table 1:** Specification of Android device.

Specification	Details
Model	Sony Xperia XZ1 G8342
Android Version	Android 9.0 (Pie)
Resolution	1080 × 1920 pixels, 16:9 ratio (424 ppi density)
Processor	Octa-core (4 × 2.45 GHz Kryo, 4 × 1.9 GHz Kryo)
Chipset	Qualcomm MSM8998 Snapdragon 835 (10 nm)
Storage	64 GB
Battery	Li-Ion 2700 mAh

**Table 2:** Location estimation error comparison in Universitas Brawijaya campus.

		Conventional *LAR*			Proposed *LAR*		
**Location**	**True Coordinate (lat., lon.)**	**Estimated Coordinate (lat., lon.)**	**Estimation Error (m)**	**ME/SD**	**Estimated Coordinate (lat., lon.)**	**Estimation Error (m)**	**ME/SD**
POI 1	−7.953868112, 112.6142459	−7.953917379, 112.6142794	6.602	8.067/1.423	−7.953867379, 112.6142524	0.716	0.990/0.274
		−7.953942448, 112.6142874	9.443		−7.953867413, 112.6142574	1.264	
		−7.953878399, 112.6143192	8.155		−7.953867133, 112.6142548	0.991	
POI 2	−7.953443107, 112.6142991	−7.953449192, 112.6143548	6.170	7.121/0.991	−7.953445192, 112.6142908	0.944	0.796/0.149
		−7.953449583, 112.6143728	8.147		−7.953445402, 112.6142937	0.647	
		−7.953449089, 112.6143628	7.046		−7.953445296, 112.6142922	0.797	
POI 3	−7.952920717, 112.6146038	−7.952947122, 112.6146455	5.453	6.119/0.851	−7.952917122, 112.6146055	0.442	0.468/0.125
		−7.952955142, 112.6146578	7.077		−7.952916687, 112.6146075	0.605	
		−7.952949549, 112.614648	5.826		−7.952918345, 112.614606	0.358	
POI 4	−7.952770084, 112.6135891	−7.952799084, 112.6136291	5.459	7.387/1.720	−7.952769084, 112.6135801	0.997	0.879/0.118
		−7.952757093, 112.6136676	8.763		−7.952769316, 112.6135822	0.762	
		−7.952751474, 112.6136587	7.938		−7.952768912, 112.6135812	0.876	
POI 5	−7.952742923, 112.6144279	−7.952785123, 112.6144789	7.319	7.477 / 0.953	−7.952739123, 112.6144229	0.694	0.601/0.091
		−7.952786095, 112.6144692	6.613		−7.952739785, 112.6144245	0.511	
		−7.952784537, 112.6144926	8.498		−7.952739426, 112.6144238	0.599	
Overall Mean Error (ME)			7.234			0.747	
Overall RMSE			7.262			0.770	

**Table 3:** Location estimation error comparison for Okayama University.

		Conventional *LAR*			Proposed *LAR*		
**Location**	**True Coordinate (lat., lon.)**	**Estimated Coordinate (lat., lon.)**	**Estimation Error (m)**	**ME/SD**	**Estimated Coordinate (lat., lon.)**	**Estimation Error (m)**	**ME/SD**
POI 6	34.68928056, 133.9227488	34.68929956, 133.9227988	5.036	6.195/1.004	34.68927456, 133.9227448	0.761	0.780/0.183
		34.68931165, 133.9228125	6.767		34.68927299, 133.9227435	0.972	
		34.68932257, 133.9228026	6.783		34.68927615, 133.9227449	0.607	
POI 7	34.68927971, 133.9233461	34.68929981, 133.9234261	7.648	8.723/1.025	34.68927261, 133.9233421	0.870	0.960/0.199
		34.68929795, 133.9234498	9.690		34.68926987, 133.9233411	1.188	
		34.68930474, 133.9234378	8.832		34.68927287, 133.9233427	0.822	
POI 8	34.68928382, 133.9196245	34.68929892, 133.9196845	5.737	7.457/1.535	34.68928159, 133.9196295	0.520	0.570/0.063
		34.68929987, 133.9197175	8.688		34.68928015, 133.9196299	0.641	
		34.68930152, 133.9197087	7.946		34.68928201, 133.9196301	0.550	
POI 9	34.68741724, 133.9125578	34.68743935, 133.9125998	4.560	6.701/1.947	34.68741784, 133.9125657	0.725	0.865/0.147
		34.68743896, 133.9126454	8.366		34.68741791, 133.9125689	1.018	
		34.68744078, 133.9124847	7.178		34.68741787, 133.9125671	0.853	
POI 10	34.69074221, 133.9203921	34.69075731, 133.9204543	5.929	7.565/2.096	34.69074341, 133.9203857	0.600	0.804/0.197
		34.69078144, 133.9204497	6.838		34.69074378, 133.9203814	0.994	
		34.69076497, 133.9204971	9.928		34.69074389, 133.9203834	0.817	
Overall Mean Error (ME)			7.328			0.796	
Overall RMSE			7.379			0.806	

## Data Availability

Data are contained within the article.
